# Paralytic Shellfish Toxin Extraction from Bivalve Meat for Analysis Using Potentiometric Chemical Sensors

**DOI:** 10.3390/bios14100487

**Published:** 2024-10-08

**Authors:** Ana Filipa R. Cerqueira, Catarina Moreirinha, Mariana Raposo, Maria Teresa S. R. Gomes, Sara T. Costa, Maria João Botelho, Alisa Rudnitskaya

**Affiliations:** 1 Centre for Environmental and Marine Studies (CESAM) and Chemistry Department, University of Aveiro, 3810-193 Aveiro, Portugal; anacerqueira@ua.pt (A.F.R.C.); catarina.fm@ua.pt (C.M.); micr@ua.pt (M.R.); mtgomes@ua.pt (M.T.S.R.G.); 2 Portuguese Institute for the Sea and Atmosphere (IPMA), 1449-006 Lisbon, Portugal; sara.dacosta@ipma.pt (S.T.C.); mjbotelho@ipma.pt (M.J.B.); 3 Interdisciplinary Centre of Marine and Environmental Research (CIIMAR), University of Porto, 4050-123 Porto, Portugal; 4 Abel Salazar Biomedical Sciences Institute, University of Porto (ICBAS), Largo Prof. Abel Salazar, 2, 4099-003 Porto, Portugal

**Keywords:** paralytic shellfish toxins, extraction, potentiometric sensors, bivalves

## Abstract

A simple and reliable methodology for the detection of paralytic shellfish toxins (PSTs) in bivalve tissues using potentiometric chemical sensors was developed. Five methods of PST extraction from mussel and oyster tissues were evaluated, including the AOAC-recommended method, which served as the reference. The main objective was to minimize the matrix effect of the extracts on the sensors’ responses and ensure efficient toxin recovery. Extraction procedures using acetic acid with heating and water yielded the highest responses from the potentiometric chemical sensors to PSTs. The highest recovery of PSTs from bivalve tissues was achieved with extraction using acetic acid and heating. Further extract purification, which is indispensable for liquid chromatography with fluorometric detection (LC-FLD) analysis, was found to be unnecessary for analysis with chemical sensors. While water extraction can also be used as a rapid and simple PST extraction method, the lower recoveries should be considered when interpreting the results. Further research is needed to identify the compounds remaining in the extracts that cause a decrease in sensor responses and to develop procedures for their elimination.

## 1. Introduction

Paralytic shellfish toxins (PSTs) are naturally occurring compounds produced by some phytoplankton species such as marine dinoflagellates and freshwater cyanobacteria. Filter-feeding invertebrates such as bivalves and gastropods can accumulate PSTs during the events of phytoplankton proliferation or Harmful Algal Blooms (HABs) [1]. These toxins pose a significant risk to human health when contaminated shellfish is ingested, causing paralytic shellfish poisoning [2]. PSTs act by blocking sodium channels in nerve cells, disrupting normal nerve signal transmission, and producing a range of neurological symptoms, including tingling, numbness, and respiratory paralysis [3]. The effective monitoring and management of PSTs in marine environments are crucial for safeguarding public health and ensuring the sustainability of the shellfish industry. Consequently, routine survey programs for marine toxins in commercial bivalve species were established worldwide, including in EU countries [4,5].

The official method for PST detection in the EU is liquid chromatography with fluorometric detection (LC–FLD) [6]. However, this method is labor-intensive and requires expensive equipment, time-consuming sample preparation, and highly skilled personnel. This underscores the need for simpler and more cost-effective probes for marine toxin detection. To address this need, several rapid testing tools including ELISA, lateral flow assays, and biosensors have been proposed [7]. In our previous work, we developed a series of potentiometric chemical sensors with plasticized PVC membranes for the detection of PSTs [8]. The detection of PSTs represent a challenge from an analytical point of view due to (i) the existence of more than 50 structural analogs; (ii) their instability under certain conditions, resulting in their transformation into each other; (iii) and the different toxicities of individual analogs, leading to changes in the total toxicity when transformations occur.

All the analytical techniques listed above rely on PST extraction from bivalve meat prior to analysis. The most extensively studied and validated extraction procedure is the one used as the reference, the AOAC method [6]. This procedure involves liquid–solid extraction in a boiling water bath using hydrochloric acid (0.1 mol L^−1^) [9] or acetic acid (1%) [10,11,12], a centrifugation step to remove precipitated proteins, followed by a clean-up step prior to the injection into the LC system. The clean-up is usually carried out by a solid-phase extraction using C18 cartridges to remove interferences (e.g., salts, lipids, and suspended particles) that can hinder the chromatographic separation. The recovery efficiency of the extraction depends on the specific toxin and matrix analyzed, with reported values varying between 50% and 120% [9,10]. The chemical conversion of PSTs during the extraction process may alter toxin profiles and, consequently, the total toxicity of the sample [4]. Heat treatment at acidic pH leads to the partial conversion of N-sulfocarbamoyl toxins into their corresponding carbamate toxins through hydrolysis at the R4 site [4,13] For example, the less toxic gonyautoxin 5 can be converted into the more toxic saxitoxin (STX) during the boiling process with a hydrochloric acid solution.

Alternative toxin extraction procedures that do not employ heating, and thus prevent toxin interconversion, have been proposed. Extraction with acetonitrile/water/formic acid (80:19.9:0.1, *v*/*v*/*v*) was used for the preparation of bivalve tissues for LC-FLD and LC-MS/MS analysis [14]. This method results in toxin recoveries comparable to the AOAC method, although the procedure is quite lengthy and cumbersome, involving the freezing of the extracts for at least 4 h and the subsequent separation of the aqueous and organic layers [15,16]. A method integrating the extraction and clean-up steps, matrix solid-phase dispersion (MSPD), has been applied to PST extraction from shellfish in [17]. The MSPD procedure involves blending homogenized bivalve tissues with C18 sorbent, packaging them into a solid-phase extraction cartridge, eluting with acetonitrile/water/formic acid (80:19.9:0.1, *v*/*v*/*v*) and extracting twice with chloroform. Then, the aqueous phase is analyzed by LC–MS/MS. This procedure is quite laborious, and, although it prevented chemical transformation of PSTs to some degree, the toxin recoveries were relatively low compared to the AOAC method.

The described PST extraction procedures prior to LC-FLD or LC-MS/MS analysis require several steps and are laborious and lengthy. Therefore, simplified toxin extraction procedures compatible with rapid, on-site, and point-of-care detection methods such as ELISA tests have also been proposed. Sample preparation procedures recommended by the test manufacturers typically involve a one-step extraction of bivalve tissues using methanol/water (80:20, *v*/*v*) [18,19], an acetate buffer [20], or water [21]. These methods are attractive for their simplicity, although data on their recovery efficiency and matrix effects are scarce. 

The objective of the present study was to develop and optimize a simple and reliable extraction procedure for detecting PSTs using potentiometric chemical sensors. To this end, oyster and mussel samples were extracted using five procedures that were evaluated with respect to matrix effects on sensor response and toxin recovery efficiency.

## 2. Materials and Methods

### 2.1. Reagents and Materials

Aniline, sodium dodecyl sulfate (SDS), sodium hydroxide, acetic acid, ammonium formate, and hydrogen peroxide were from Merck Life Science S.L. (Alges, Portugal); and hydrochloric acid and sulfuric acid were from Panreac Química S.L.U. (Barcelona, Spain); tetrahydrofuran (Chromasolv) was from Fisher Scientific Lda. (Porto, Portugal). Methanol and acetonitrile were from Riedel-de Haën (VWR International, Carnaxide, Portugal). High-molecular-weight polyvinyl chloride (PVC), dibutyl phthalate (DBP), potassium tetrakis(4-chlorophenyl)borate (KTClPhB) and ionophores (octadecyl 4-formylbenzoate (IP1), and calix [4]arene-25,26,27,28-tetrol (IP2)) were from Fluka (Merck Life Science S.L., Alges, Portugal). All reagents were analytical or liquid chromatography grade.

Certified standard solutions of PSTs, namely decarbamoyl saxitoxin (dcSTX), saxitoxin (STX), gonyautoxins 2 and 3 (GTX2&3), and N-sulfocarbamoyl gonyautoxins 2 and 3 (C1&2) were certified reference materials from the CIFGA Laboratories (Lugo, Spain).

Screen-printed electrodes (SPE) with gold-working and auxiliary electrodes and a silver reference electrode were from Metrohm DropSens (Oviedo, Spain). A platinum sheet counter electrode and an Ag/AgCl (3 mol L^−1^ KCl) reference electrode were from Metrohm (Herisau, Switzerland). Ultrapure water produced by Merck Millipore Water System (18 MΩcm-1) was used for all solution preparation and sensor washing.

### 2.2. Preparation of Bivalve Extracts

Optimization of the PST extraction from bivalve’s tissues was performed by comparing five adapted procedures known to be used for the determination of PSTs by liquid chromatography or ELISA assays. The procedure, the tested matrices, and the respective reference are listed in Table 1. All bivalve extracts were stored at −20 °C prior to analysis.

**1a.** The official AOAC method consisting of two steps, an acidic extraction and a clean-up, was used for the preparation of bivalve extracts [6]. The acidic extraction was performed by adding 3 mL of 1% acetic acid to 5 g of bivalve tissue, which was then heated in a boiling water bath for 5 min. After cooling the extracts under tap water, they were centrifuged at 3600× *g* for 10 min at room temperature. The supernatant was collected, and the tissue residue was subjected to a second extraction with 3 mL of 1% acetic acid, this time without heating. The two supernatants were combined, and the final volume was adjusted to 10 mL with ultrapure water, forming the acidic extract. Subsequently, 1 mL of the extract was passed through a solid phase extraction C18 cartridge (500 mg/3 mL, Supelclean, Merck Life Science S.L. (Alges, Portugal), and the final volume was adjusted to 4 mL with ultrapure water. Prior to analysis by potentiometric sensors, the pH of the cleaned extracts was adjusted to 7 with 1 mol L^−1^ Tris base solution.

**1b.** Mussel extracts were prepared according to the official AOAC method as described above (1a), but the clean-up using C18 solid phase extraction cartridges was carried out twice.

**2.** Only the first step of the official sample preparation method described above, i.e., extraction with acetic acid, was implemented. The extracts were filtered using polytetrafluoroethylene polymer (PTFE) filters (0.2 µm, 17 mm) prior to analysis.

**3a.** Toxin extraction with water was performed according to the sample preparation method recommended for Reveal® rapid ELISA test [20]. In summary, 5 g of edible bivalve tissues were homogenized with 3 mL of ultrapure water using an ultra-turrax for 30 s, centrifuged at 3600× *g* for 10 min, and the supernatant was saved. An additional 3 mL of ultrapure water was added to the mussels’ tissue residue, vortexed for 3 min, and re-centrifuged for another 10 min at 3600× *g*. The supernatants from both extractions were combined, and the total volume was brought to 10 mL with ultrapure water. The extracts were filtered using regenerated cellulose (RC) filters (0.2 µm, 17 mm) prior to analysis.

**3b.** Water extracts prepared as described above in 3a were subjected to a clean-up step using a C18 cartridge, as described for the extracts in 1a.

**4.** Extraction with methanol was carried out according to the sample preparation method recommended for Beacon plate kit immunoassay [17]. Homogenized soft tissues of bivalves (1 g) were mixed with 1 mL of methanol/water (80:20, *v*/*v*) using an ultra-turrax. The mixture was centrifuged for 10 min at 3000× *g* and supernatant were collected. A total of 0.8 mL methanol/water (80:20, *v*/*v*) was added to the bivalve tissue residues, centrifuged for 10 min at 3000× *g*, and joined with the first portion of the supernatant. The collected supernatants were diluted to 2 mL with methanol/ water (80:20, *v*/*v*) and filtered through RC filters (0.2 µm, 17 mm) prior to analysis.

**5.** Extract preparation was carried out according to the procedure described in [14].

In brief, 5 g of bivalve tissue were homogenized with 10 mL of a mixture of acetonitrile/water (80:20, *v*/*v*) with added formic acid (0.1%) at 10,000 rpm for 5 min using an ultra-turrax, while the extract was cooled in ice water. The homogenate was centrifuged at 3500 rpm for 15 min, and the supernatant was stored in a freezer at −20 °C for 4 h. Freezing the supernatant resulted in its separation into two layers: a frozen aqueous lower layer, and a liquid organic upper layer. After removal from the freezer, the upper layer was decanted and discarded. The lower aqueous layer was then reduced to less than 3 mL by air flow in a heating block at 40 °C and subsequently adjusted to 3 mL with ultrapure water.

### 2.3. Quantification of PSTs in Bivalve Extracts by LC-FLD

Quantification of PSTs in bivalve extracts prepared using the 5 procedures described above was based on the official AOAC method [6]. Determination of N-sulfocarbamoyl and decarbamoyl compounds that are predominant in the *Gymnodinium catenatum* toxin profile was carried out according to the modified PST oxidation [21]. Details of the oxidation procedure and chromatographic conditions used for PSTs’ quantification in the bivalve extracts were described in [21].

### 2.4. PSTs Recovery Study

Recoveries and losses of the used analytical procedures were assessed following the Quevauviller and Morabito methodology [22]. Mussel and oyster matrices previously confirmed as PST-free samples were used. Both matrices were spiked in triplicate with the addition of dcSTX and STX certified standard solutions to uncontaminated matrices; an equilibration time of 12 h was used before extraction procedures following methods 1a and 3a (Table 1). Before standard solutions addition, matrices were autoclaved for enzyme inactivation (121 °C, 15 min). Spike concentrations were 1.750 µg g^−1^ (0.66 µmol L^−1^) for dcSTX and 2.600 µg g^−1^ (0.87 µmol L^−1^) for STX. Toxin quantification was carried out using reference method for PSTs in bivalve extracts, LC-FLD, as described in Section 2.3.

### 2.5. Sensor Fabrication

Commercially available SPE were used for preparation of potentiometric sensors according to the procedure developed previously [8]. The surface of the SPE working electrode (gold or carbon) was thoroughly cleaned with ethanol and water, followed by electrochemical cleaning by cycling the potential between −0.2 and +1.2 V at a scan rate of 50 mV s^−1^ in 50 mmol L^−1^ sulfuric acid for three cycles. Preparation of the solid inner contact was carried out by electropolymerizing aniline from a deaerated aqueous solution containing 50 mmol L^−1^ aniline and 1 mol L^−1^ hydrochloric acid and 0.1 mol L^−1^ SDS by cycling potential for 80 cycles between −0.23 and +0.85 V at 50 mV s^−1^. Sensors were washed with deionized water, conditioned for 2 h in 0.1 mol L^−1^ hydrochloric acid, and air dried.

Membrane cocktails were prepared by dissolving PVC (33% *w*/*w*), DBP (65% *w*/*w*), ionophore (1.5% *w*/*w*), and KTClPhB (0.5% *w*/*w*) in tetrahydrofuran. Two ionophores were used: octadecyl 4-formylbenzoate (IP1) and calix [4]arene-25,26,27,28-tetrol (IP2). An amount of 10 µL of membrane mixture was drop casted on the solid contact of the SPE and left to dry overnight at room temperature. Before use, the sensors were conditioned in ultrapure water for 2 h.

Electrochemical experiments were performed with a multichannel Bipotentiostat/Galvanostat/EIS μStat-i MultiX (Metrohm-Dropsens, Oviedo, Spain). External platinum sheet counter electrode and Ag/AgCl (3 mol L^−1^ KCl) reference electrode were used.

### 2.6. Solution Preparation and Potentiometric Measurements

Calibration solutions were prepared by diluting certified standard dcSTX, STX, GTX2&3, and C1&2 solutions in 0.25 mmol L^−1^ Tris HCl buffer (pH 7) or in PST-free bivalve extracts. pH of the acidic extracts was adjusted to 7 with Tris-base 1 mol L^−1^ solution. The concentration of the toxins ranged from 0.17 to 4.8 µmol L^−1^. 

A custom-built high-input impedance digital voltmeter (Sensor Systems LLC., St. Petersburg, Russia) was used for potentiometric measurement. The voltmeter was connected to a PC for data acquisition. Sensor potentials were measured against the pseudo-reference electrode of the SPE. A 35 µL drop of solution was applied to the surface of the sensor chip, and the potential was recorded after 5 min. Between measurements, sensors were washed with copious amounts of deionized water.

## 3. Results and Discussion

Two groups of PSTs corresponding to the typical toxin profiles observed in the bivalve tissues after exposure to *Alexandrium* spp. and *G. catenatum* were selected for the development of an experimental procedure for their detection using potentiometric sensors. Two representative toxins of each group were selected: STX and GTX2&3 for the *Alexandrium* spp. profile and dcSTX and C1&2 for the *G. catenatum* profile [4]. Using several PST analogs for testing the extraction procedures was deemed necessary due to the different selectivity of the sensors [8]. Furthermore, extraction recoveries among toxin analogs can vary. Notably, high recoveries were reported for dcSTX, while recoveries for STX tend to be lower and less consistent among the studies [23].

**Figure 1:**
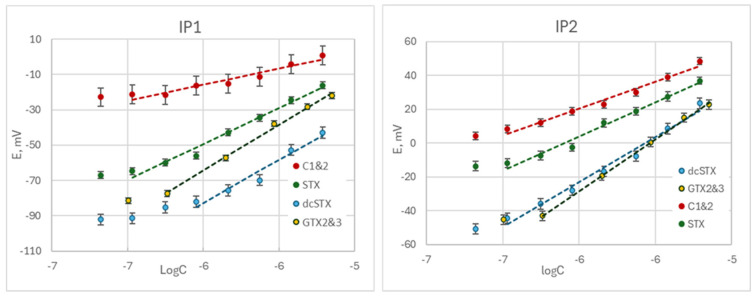
Responses of the sensors IP1 and IP2 to four PSTs, dcSTX, STX, C1&2, and GTX2&3 in the solutions prepared in Tris HCl buffer (0.25 mmol L^−1^, pH7). Standard deviation of three measurements with the same sensor and regression lines are shown.

The sensor compositions used for the assessment of the PST extraction procedures were selected based on our previous work [8]. A series of potentiometric chemical sensors with solid inner contact and plasticized polyvinylchloride membranes containing different ionophores were characterized in the individual solutions of eight PSTs, including carbamate, decarbamoyl, and N-sulfocarbamoyl analogs [8]. All developed sensors displayed cross-sensitivity to all toxins with varying sensitivity and selectivity. Based on these results, two sensor compositions were selected for the extraction procedures’ tests to ensure a high response to all four PSTs. Octadecyl 4-formylbenzoate (IP1) exhibited a high sensitivity for dcSTX, STX, and GTX2&3 with the slopes of 60, 47, and 56 mV/logC, and a low sensitivity to C1&2 with the slope of 20 mV/logC. Calix [4]arene-25,26,27,28-tetrol (IP2) exhibited a higher sensitivity for C1&2 with the slope of 29 mV/logC and a lower sensitivity to STX with the slope of 36 mV/logC compared to the IP1. IP2 also displayed a high sensitivity to dcSTX and GTX2&3 with slopes of 57 and 61 mV/logC, respectively. The responses of two sensors to dcSTX, STX, C1&2, and GTX2&3 in the solutions prepared in Tris HCl buffer (pH 7) are shown in Figure 1.

PST extraction procedures were compiled based on data in the literature with some modifications (Table 1). The AOAC recommended procedure (1a) was used as a reference. First, the measurements were conducted using sensor IP1 in mussel extracts prepared using all procedures listed in Table 1 and spiked with dcSTX standards. The preliminary tests revealed that extracts prepared using organic solvents according to methods 4 and 5 damaged the sensing membranes, while method 5 was also found to be too cumbersome for practical use. Therefore, these two methods were excluded from further experiments.

**Figure 2:**
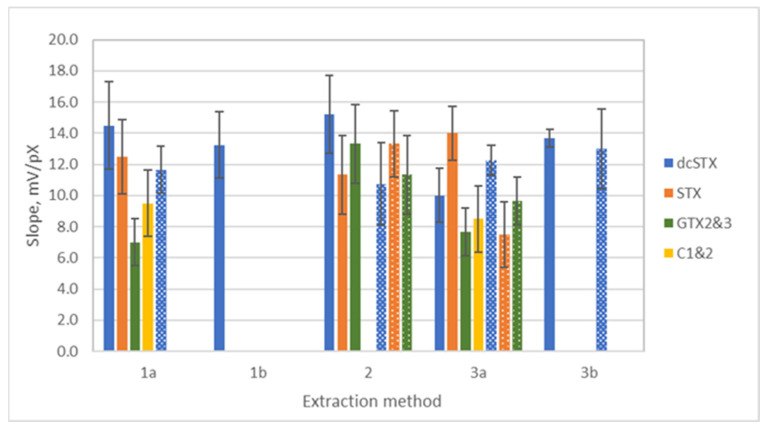
Slopes of the electrode function of the sensor IP1 in the solutions of dcSTX, STX, and GTX2&3 and sensor IP2 in the solutions of C1&2 prepared in mussel (solid columns) and oyster (patterned columns) extracts. Mean values (*n* = 3) with standard deviations are shown.

Sensor IP1 exhibited a response to dcSTX in both mussel and oyster extracts prepared using procedures 1a, 1b, 2, 3a, and 3b (Figure 2). The calibration curves of the sensors IP1 and IP2 in the toxin solutions in extracts are shown in Figure S1. The slopes of the electrode function were lower than those observed in buffer solutions, which were close to the theoretical value for a single-charged cation (Figure 1). The decrease in sensitivity observed in bivalve extracts compared to buffer solutions was reported for other analytical techniques as well, e.g., LC-FLD [21]. However, no changes in the linear working range and detection limits were observed in the bivalve extracts compared to the buffer solutions [8]. The differences between the slopes of the electrode function of the sensors IP1 to dcSTx, STX, and GTX2&3 in the extracts 1a, 2, and 3a, and the sensor IP2 to C1&2 in the extracts 1a and 3a, were not statistically significant according to the ANOVA analysis (*p*-value = 0.05) for both mussel and oyster extracts with the exception of the sensor IP1 response to dcSTX, which was lower in the mussel extract 3a compared to extract 2 (*p* = 0.02). Thus, the clean-up using a C18 cartridge is presumably not necessary for the sample preparation for measurements with sensors, as compounds removed by this method do not interfere with the sensor response. In an attempt to minimize matrix effects, the extracts prepared using methods 1a and 3a were submitted to an additional clean-up step using a C18 cartridge (extracts 1b and 3b), and calibration measurements were carried out with sensor IP1 in dcSTX solutions. The additional clean-up step did not result in significantly different slope values (*p* = 0.5). Therefore, simpler preparation procedures involving fewer steps, namely 2 and 3a, were used for further experiments.

In the next step, the calibration measurements with sensors IP1 and IP2 were carried out in mussel and oyster extracts 1a, 2, and 3a spiked with STX, GTX2&3, and C1&2. The sensors responded to all toxins in all measured extracts except C1&2, for which only responses in mussel extracts 1a and 3a were obtained (Figure 2). Similar to dcSTX, the sensor responses to other PSTs in bivalve extracts were lower than those observed in buffer solutions and were not statistically different between preparation methods, except the responses to GTX2&3 in mussel extracts. The GTX2&3 response in mussel extract 2 was statistically higher (*p*-value = 0.02) compared to the mussel extracts 1a and 3a.

**Figure 3:**
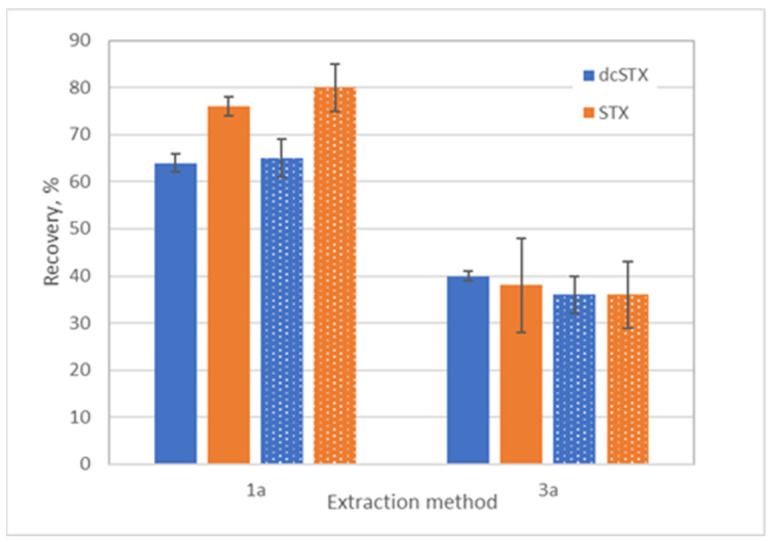
Recoveries of PSTs (%) for mussel (solid columns) and oyster (patterned columns) matrices prepared using procedures 1a and 3a (Table 1) and determined by LC-FLD. Mean values (*n* = 3) with standard deviations are shown.

After having selected methods 2 and 3a for potentiometric toxin detection, the efficiency of the sample preparation procedures in quantitatively extracting toxins from the bivalve tissues was evaluated. Recovery tests were conducted in uncontaminated mussel and oyster matrices spiked with dcSTX and STX standards, and toxin extracts were prepared using methods 1a and 3b and then analyzed by LC-FLD. It is important to note that method 2 is a simplified version of reference procedure 1a, which does not involve the extract clean-up using a C18 cartridge. As clean-up is required for PSTs’ quantification in bivalve extracts using LC-FLD, the extracts prepared using methods 2 and 3a needed to be submitted to clean-up using a C18 cartridge. Thus, the resulting extracts corresponded to the reference AOAC method 1a and method 3b, respectively. The recoveries using reference method were ca. 65% for dcSTX and 80% for STX, while recoveries using extraction with water were lower, varying between 36 and 40% for both toxins (Figure 3).

## 4. Conclusions

An experimental procedure for the detection of PSTs in bivalve meat using potentiometric chemical sensors was developed. Among the studied sample preparation methods, extraction with acetic acid with heating (procedure 2) and extraction with water (procedure 3) ensured the highest sensor response to four studied PSTs (dcSTX, STX, C1&2, and GTX2&3) in both mussel and oyster extracts. Clean-up using a C18 cartridge was found to be unnecessary for the analysis with chemical sensors. Since the highest recovery of PSTs from bivalve tissues was obtained using procedure 2, this method can be recommended for sample preparation for analyses using sensors. Extraction with water (procedure 3a) can also be used as a rapid and simple method of PST extraction, although lower toxin recoveries should be taken into account when interpreting the results. Further research is necessary to identify compounds that remain in the extracts after sample preparation and cause a decrease in the sensor response, as well as to develop procedures for their elimination.

## Figures and Tables

**Table 1:** Sample preparation procedures for PST extraction from bivalve tissues. ME—mussel extracts, OE—oyster extracts.

Extract		Procedure	Ref.
1a	ME, OE	Acetic acid 1% + clean-up with C18	[1]
1b	ME	Acetic acid 1% + clean-up with C18 twice	
2	ME, OE	Acetic acid 1%	
3a	ME, OE	Water	[20]
3b	ME, OE	Water + clean-up with C18	
4	ME	Methanol/water (80:20 *v*/*v*)	[17]
5	ME	acetonitrile/water/formic acid (80:19.9:0.1, *v*/*v*/*v*)	[14]

## Data Availability

Data are contained within the article and Supplementary Materials.

## Supplementary Materials

The following supporting information can be downloaded at: https://www.mdpi.com/article/10.3390/bios14100487/s1, Figure S1: Responses of the sensors IP1 (a–c) and IP2 (d) to four PSTs, dcSTX, STX, C1&2 and GTX2&3 in the mussel (solid symbols) and oyster (open symbols) extracts prepared using different extraction procedures title.
