# Supplementary material for: Paralytic Shellfish Toxin Extraction from Bivalve Meat for Analysis Using Potentiometric Chemical Sensors
Source: Biosensors (Basel). 2024 Oct 8;14(10):487. doi: 10.3390/bios14100487 (PMC11506007; doi:10.3390/bios14100487)

biosensors

Paralytic Shellfish Toxin Extraction from Bivalve Meat for Analysis Using Potentiometric Chemical Sensors

Ana Filipa R. Cerqueira [1], Catarina Moreirinha [1], Mariana Raposo [1], Maria Teresa S. R. Gomes [1], Sara T. Costa [2,3,4],
Maria João Botelho [2,3] and Alisa Rudnitskaya [1,*]

[1] CESAM and Chemistry Department, University of Aveiro, 3810-193 Aveiro, Portugal;
anacerqueira@ua.pt (A.F.R.C.); catarina.fm@ua.pt (C.M.); micr@ua.pt (M.R.); mtgomes@ua.pt (M.T.S.R.G.)

[2] IPMA, Portuguese Institute for the Sea and Atmosphere, 1449-006 Lisbon, Portugal;
sara.dacosta@ipma.pt (S.T.C.); mjbotelho@ipma.pt (M.J.B.)

[3] CIIMAR, Interdisciplinary Centre of Marine and Environmental Research, University of Porto,
4050-123 Porto, Portugal

[4] ICBAS, Abel Salazar Biomedical Sciences Institute, University of Porto, Largo Prof. Abel Salazar, 2,
4099-003 Porto, Portugal

* Correspondence: alisa@ua.pt

Fig. 1S. Responses of the sensors IP1 (a-c) and IP2 (d) to four PSTs, dcSTX, STX, C1&2 and GTX2&3 in the mussel (solid symbols) and oyster (open symbols) extracts prepared using different extraction procedures (see table 1 for details). Standard deviation of three measurements with the same sensor and regression lines are shown.

a

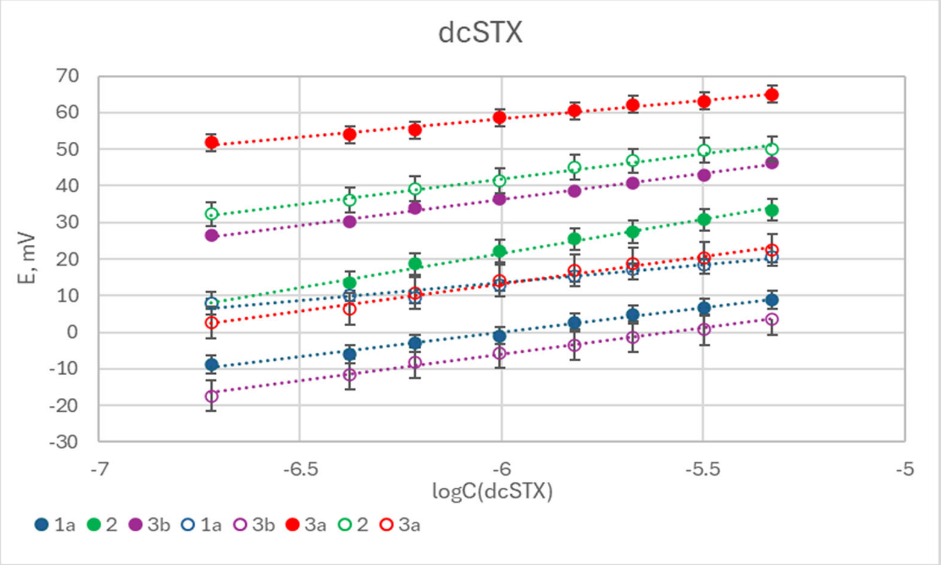

b

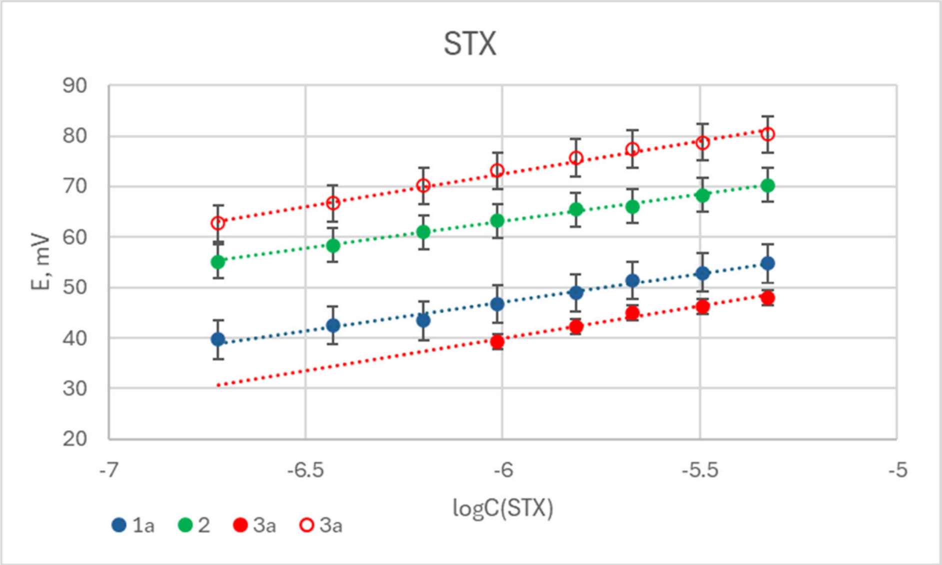

biosensors

MDPI

c

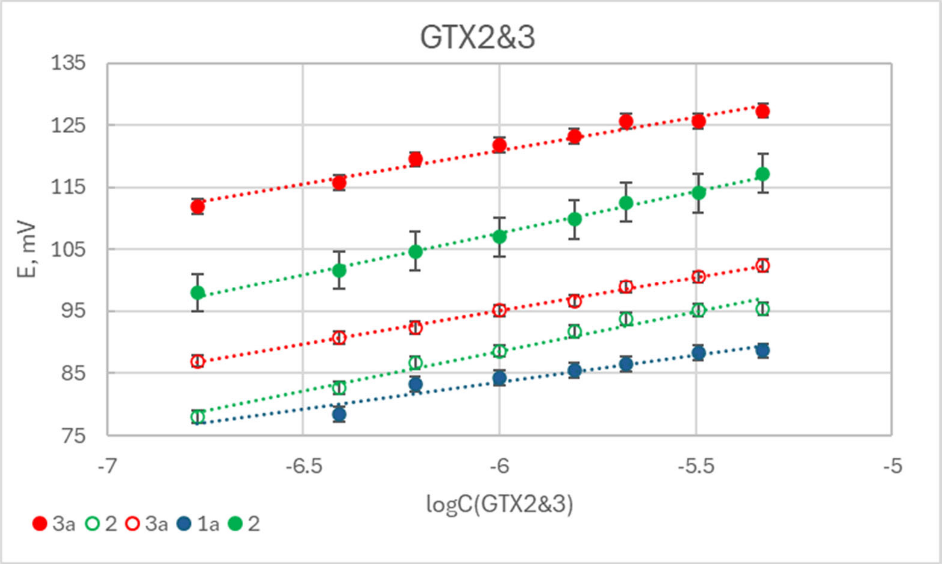

d

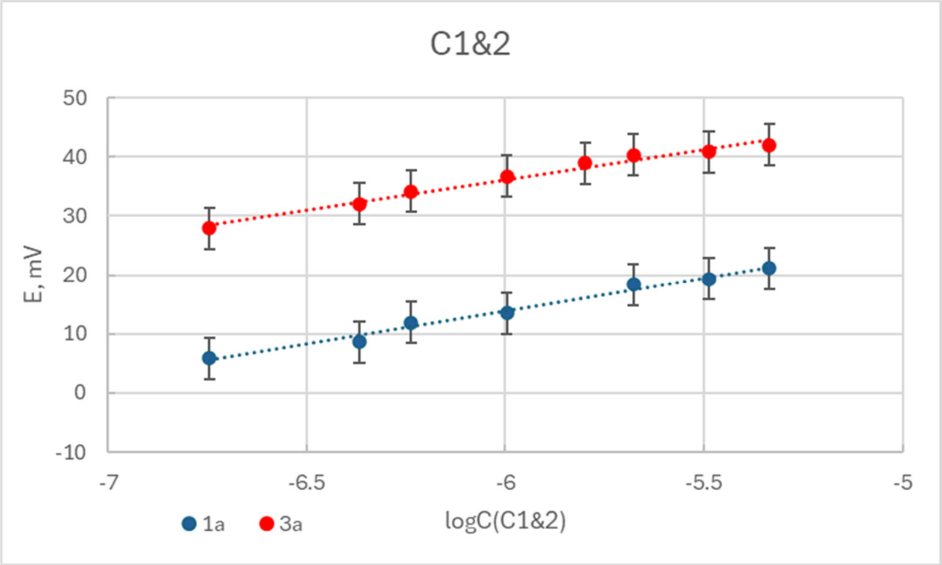

Supplement: Supplementary file 1 [file biosensors-14-00487-s001.zip › biosensors-3166547-supplementary.pdf]
